# Estimated enterolignans, lignan-rich foods, and fibre in relation to survival after postmenopausal breast cancer

**DOI:** 10.1038/bjc.2011.374

**Published:** 2011-09-13

**Authors:** K Buck, A K Zaineddin, A Vrieling, J Heinz, J Linseisen, D Flesch-Janys, J Chang-Claude

**Affiliations:** 1 Unit of Genetic Epidemiology, Division of Cancer Epidemiology, German Cancer Research Center (DKFZ), Im Neuenheimer Feld 581, Heidelberg, Germany; 2 Department of Cancer Epidemiology/Clinical Cancer Registry, University Cancer Center Hamburg (UCCH), Martinistrasse 52, 20246 Hamburg, Germany; 3 Institute of Epidemiology, Helmholtz Center Munich, Ingolstädter Landstrasse1, 86746 Neuherberg, Germany; 4 Department of Medical Biometry and Epidemiology, Center for Experimental Medicine, University Medical Center Hamburg-Eppendorf, Martinistrasse 52, 20246 Hamburg, Germany

**Keywords:** phyto-oestrogens, enterolactone, seeds, fibre, postmenopausal breast cancer prognosis, survival

## Abstract

**Background:**

Lignans – oestrogenic substances present in various foods – are associated with postmenopausal breast cancer risk, but not much is known regarding their effects on survival.

**Methods:**

In a follow-up study of 2653 postmenopausal breast cancer patients diagnosed between 2001 and 2005, vital status and causes of death were verified through end of 2009. Hazard ratios (HRs) and 95% confidence intervals (CIs) for estimated enterolignans, lignan-rich foods, and dietary fibre in relation to overall survival (OS) and breast cancer-specific survival (BCSS) were assessed using Cox proportional hazards models stratified by age at diagnosis and adjusted for prognostic/confounding factors.

**Results:**

Median follow-up time was 6.4 years, and 321 women died, 235 with breast cancer. High estimated enterolactone and enterodiol levels were associated with significantly lower overall mortality (highest quintile, HR=0.60, 95% CI=0.40–0.89, *P*_Trend_=0.02 and HR=0.63, 95% CI=0.42–0.95, *P*_Trend_=0.02, respectively). Fibre intake was also associated with a significantly lower overall mortality. Differentiated by median fibre intake, associations with estimated enterolignans were still evident at low but not high fibre intake. There was no effect modification by oestrogen receptor status and menopausal hormone therapy.

**Conclusion:**

Postmenopausal breast cancer patients with high estimated enterolignans may have a better survival.

Lignans are biphenolic compounds in plant foods with a structure similar to oestrogens. They are the major source of phyto-oestrogens in Western populations and are primarily present in fibre-rich foods (such as seeds, grains, vegetables, and fruits) (Adlercreutz, 2007; Sonestedt and Wirfält, 2010). In the human gut, plant lignans are converted by intestinal bacteria to the enterolignans, enterolactone and enterodiol, which are bioactive and are subsequently absorbed (Adlercreutz, 2007).

There are several mechanisms by which enterolignans may protect against cancer. They were shown to have weak oestrogenic activity and may bind to oestrogen receptors (ERs) (Mueller *et al*, 2004; Penttinen *et al*, 2007). Moreover, they may exert effects via oestrogen receptor-independent mechanisms, such as inhibition of tumour growth and angiogenesis, as well as stimulation of apoptosis and of sex hormone-binding globulin production (SHBG) (Adlercreutz *et al*, 1987; Power *et al*, 2006; Adlercreutz, 2007; Mense *et al*, 2008; Saarinen *et al*, 2008, 2010).

Lignans have been associated with postmenopausal breast cancer risk; however, the results were inconsistent. Two recent meta-analyses found lignans to be associated with a small risk reduction only in postmenopausal but not in premenopausal women based on studies in Eastern and Western countries (Velentzis *et al*, 2009; Buck *et al*, 2010).

There is limited epidemiological evidence on the potential effects of lignans with respect to breast cancer prognosis. Two studies, conducted in the United States, assessed the association of dietary plant lignans with survival in postmenopausal breast cancer patients (Fink *et al*, 2007; McCann *et al*, 2009). One study reported that a higher dietary lignan intake was associated with a better prognosis (McCann *et al*, 2009), whereas the other found no association (Fink *et al*, 2007). Neither study examined the association of estimated enterolignans with survival.

Dietary fibre and a diet rich in fibre has been (inconsistently) associated with a better survival after breast cancer (Holmes *et al*, 1999; Kroenke *et al*, 2005; Kellen *et al*, 2009). As lignans originate partly from fibre complex in foods, it is still unclear whether associations observed with fibre-rich foods are attributed to fibre or enterolignans or both.

Thus, we investigated the association of estimated amounts of enterolignans (enterolactone and enterodiol), dietary intake of lignan-rich foods (seeds, bread, vegetables, and fruit), and dietary fibre intake with overall and breast cancer-specific survival (BCSS) in a large German cohort of postmenopausal breast cancer patients. Furthermore, we examined whether this association is modified by hormone receptor status of the tumours and menopausal hormone therapy use at diagnosis.

## Materials and methods

### Study population

Our study population comprised breast cancer patients aged 50–74 years who participated in a population-based case–control study carried out in two German study regions (Hamburg and Rhein-Neckar-Karlsruhe (RNK)) (Flesch-Janys *et al*, 2008) and were followed up until the end of 2009. The patients were diagnosed with a histologically confirmed primary invasive or *in situ* breast tumour between 1 January 2001 and 30 September 2005 in Hamburg and between 1 August 2002 and 31 July 2005 in the RNK region and were identified through participating clinics and the Cancer Registry of Hamburg. Postmenopausal status was defined by one or more of the following criteria: last menstrual bleeding at least 12 months before diagnosis, bilateral oophorectomy, or >55 years with unclear menopausal status due to hysterectomy or hormone use. We included 2668 postmenopausal patients who completed a food frequency questionnaire (FFQ) and did not have missing information on cancer before the breast cancer diagnosis and were not previously diagnosed with cancer (except for *in situ* carcinoma, and basal and squamous cell skin carcinoma).

At recruitment, in-person interviews were performed to collect information on demographic, anthropometric, socioeconomic, and lifestyle factors. Moreover, information on suggested and established breast cancer risk factors and possible prognostic factors was obtained. Clinical and pathological records were used to collect information on further prognostic factors.

The study was approved by the ethics committees of the University of Heidelberg, the University of Hamburg, and the Medical Board of the State of Rheinland-Pfalz, and conducted in agreement with the Helsinki declaration. All participants provided written informed consent at recruitment as well as during follow-up.

### Dietary data

At recruitment, a self-administered validated 176-items FFQ was completed by the patients, which recorded the nutritional habits one year before diagnosis. This FFQ was similar to the one used in the European Prospective into Cancer and Nutrition (EPIC) study, and nutrient intake (e.g., dietary fibre) was calculated using the German food composition table Bundeslebensmittelschluessel II.3 (Bundesinstitut für Gesundheitlichen Verbraucherschutz und Veterinärmedizin) (Bohlscheid-Thomas *et al*, 1997a, 1997b). For each food item, the information about portion size and consumption frequency was used to calculate intakes in grams per day (g day^–1^). The two enterolignans, enterolactone and enterodiol, were estimated from data on incubation with human faeces, in which the bioavailable enterolignans were calculated per 100 g of ingested foods (Thompson *et al*, 1991; Linseisen *et al*, 2004).

Two lignan-rich food items, sunflower-/pumpkinseeds and sesame/flaxseeds, were added to the original EPIC-FFQ for this specific study. Mean daily intake in g day^–1^ was estimated for each food item.

A top and bottom energy cutoff of 0.5% was used, as the data were considered unreliable in extreme ranges of energy intake (energy range: 425–4577 kcal day^–1^), resulting in 2653 patients finally included in the statistical analyses.

### Outcome definition

Vital status of all patients was ascertained via local population registries through the end of 2009, and causes of death were verified by death certificates and coded based on ICD-10 classifications. The end points of interest were overall survival (OS) and BCSS. The OS included death from any cause whereas for BCSS, deaths from breast cancer (coded as ICD-10 C50) were events of interest and deaths from other causes were censored at date of occurrence. Women without an event of interest were censored either at date of last information or end of 2009. Median follow-up time was calculated as time between diagnosis and the event of interest or censoring using the reverse Kaplan–Meier estimation (Schemper and Smith, 1996).

### Statistical analysis

Differences in baseline characteristics between deceased and non-deceased patients were tested using the *χ*^2^-statistic. The Kruskal–Wallis test was used to evaluate differences in the levels of enterolignans and lignan-rich foods between deceased and non-deceased patients. Spearman's correlation coefficients were used to correlate enterolignan intake with other dietary intake variables.

Cox proportional hazards models were used to estimate hazard ratios (HRs) for OS and BCSS, and their 95% confidence intervals (CIs) associated with enterolignans and fibre (in quintiles) using the lowest quintile as reference group. For the assessment of seed intake and prognosis, non-consumers were used as the reference category, and were compared with low consumers (intake below median consumption of the consumers) and high consumers (intake above and equal to median consumption of the consumers). Total bread (white, brown, and whole-grain), vegetable, and fruit intake were categorised into tertiles using the lowest tertile as reference group.

All analyses were stratified by age at diagnosis (in 1-year categories) and adjusted for the established prognostic factors tumour size (<2 cm, 2–5 cm, ⩾5 cm, growth into chest wall, neoadjuvant chemotherapy-treated carcinoma, *in situ* carcinoma), nodal status (0, 1–3, 4–9, ⩾10, neoadjuvant chemotherapy-treated carcinoma, *in situ* carcinoma), metastasis (yes, no, *in situ* carcinoma), grade (1+2, 3+4, *in situ* carcinoma, neo-adjuvant chemotherapy-treated carcinoma), and ER/progesterone receptor (PR) status (*in situ* carcinoma, ER+/PR+, ER+/PR− or ER−/PR+, ER−/PR−, neoadjuvant chemotherapy-treated carcinoma). Further covariates for the multivariable regression analyses were determined using a backward elimination procedure based on *P*<0.05 in the likelihood ratio test for the covariate or a >10% change in HR for variable of interest. Thus, the models additionally included breast cancer detection type (physician-detected by clinical examination/mammography/ultrasound, self-detected by palpation/secretion/pain), use of menopausal hormone therapy at diagnosis (current, never/past), diabetes (yes, no), and study centre (Hamburg and RNK). Additionally, energy intake (kcal day^–1^, continuous) was included as a confounder in the multivariable regression models. The HER2-neu status, cardiovascular diseases, alcohol consumption, smoking habits, occupational status, type of surgery, phyto-oestrogen supplementation, adult BMI, physical activity, and further dietary variables (i.e., vitamin C, fat, carbohydrate, protein, bread, vegetable, and fruit intake), as well as hormonal, chemo-, and radiotherapy did not affect the risk estimates in the Cox models. Potential confounding by fibre was assessed by additional adjustment of the models of enterolignans for dietary fibre intake (continuous). Similarly, the model for dietary fibre was additionally adjusted for enterolactone levels (continuous). Additionally, subgroup analyses by low and high dietary fibre intake (<median *vs* ⩾median) were performed. Tests for trend of the HRs were performed using continuous values of the variables.

To assess the functional form of the continuous variables in the multivariable Cox models, fractional polynomials were applied. The models with the best –2 log likelihoods were selected (Royston *et al*, 1999), resulting in a linear association between the log HR and exposure variables for OS and BCSS. The test of Grambsch and Therneau (Grambsch and Therneau, 1994) was used to test the proportional hazards assumption and was found to hold for all analyses.

Effect modification was evaluated using the Q-statistic. Sensitivity analyses were performed by median time between diagnosis of breast cancer and date of FFQ completion, and by exclusion of phyto-oestrogen (isoflavone-containing) supplement users. Moreover, a subgroup analysis was conducted for patients with early-stage breast cancer (stage I–IIIa). Possible effect modification was assessed using analyses stratified by ER status of the tumour (positive *vs* negative), ER/PR status of the tumour (ER- or PR-positive *vs* ER-/PR-negative), and menopausal hormone therapy use at diagnosis (current *vs* past/never).

For all analyses, two-sided *P*-values <0.05 were considered significant. No adjustment for multiple testing was performed because of the exploratory character of this study. All analyses were performed using R version 2.9.2 (R Development Core Team, Vienna, Austria) and SAS version 9.2 (SAS Institute, Cary, NC, USA).

## Results

### Baseline characteristics

Selected baseline characteristics of postmenopausal breast cancer patients are presented in Table 1. Median age at diagnosis of the patients was 63 years (interquartile range=9 years). Compared with the non-deceased patients, those who died within the follow-up period were older, were more likely to have higher adult BMI, larger tumours, more lymph node involvement, metastases, higher grade, ER-/PR-negative tumours, diabetes, self-detected tumours, and HT use at diagnosis. Deceased patients also had significantly lower dietary intake of sunflower-/pumpkinseeds, fibre, vegetables, and fruit, as well as lower exposure to estimated enterolignans compared with non-deceased patients (all *P*<0.05; Table 2).

Total estimated enterolignans correlated positively with dietary sunflower-/pumpkinseed and sesame/flaxseed intake (correlation of 0.27 and 0.26, respectively), and with fibre intake (correlation of 0.73). Patients with higher enterolignan exposure had higher energy, carbohydrates, protein, fat, potato, bread, vegetable, and fruit intake (data not shown).

### Prognostic association of enterolignans and lignan-rich foods

During a median follow-up time of 6.4 years, a total of 321 deaths occurred, of which 235 were due to breast cancer. Further causes of death included other cancers (*n*=41), cardiovascular diseases (*n*=22), and other causes (*n*=23).

Higher estimated levels of enterolactone, enterodiol, and dietary fibre were associated with significantly reduced overall mortality, with significantly reduced adjusted HRs for the highest compared with the lowest quintile (HR=0.60, 95% CI=0.40–0.89, *P*_Trend_=0.02, HR=0.63, 95% CI=0.42–0.95, *P*_Trend_=0.02, and HR=0.52, 95% CI=0.32–0.82, *P*_Trend_=0.01, respectively; Table 3). Multivariable regression modelling using fractional polynomials confirmed linear associations between exposure variables and OS. Associations of enterolignans and fibre with BCSS were similar to that with OS; however, the HRs were not statistically significant.

Additional adjustment for dietary fibre intake in the assessments of estimated enterolignans with respect to OS did not substantially alter the associations; however, they became nonsignificant (Supplementary Table 1). The association of dietary fibre with overall mortality also became nonsignificant after adjustment for enterolactone (Supplementary Table 1). When differentiated according to median dietary fibre intake, higher levels of both enterolactone and enterodiol were still significantly associated with lower overall mortality for the low fibre intake but not high fibre intake group, although the HRs for the highest quintiles were nonsignificant (highest quintile for enterolactone HR=0.32, 95% CI=0.10–1.04, *P*_Trend_=0.03 and HR=1.25, 95% CI=0.44–3.58, *P*_Trend_=0.08, respectively, and enterodiol HR=0.48, 95% CI=0.21–1.07, *P*_Trend_=0.01 and HR=1.08, 95% CI=0.38–3.04, *P*_Trend_=0.05, respectively; Table 4).

For the lignan-rich foods, there was no trend in association by level of consumption although the HRs for low *vs* no consumption were significant for sunflower-/pumpkinseeds (0.74, 95% CI=0.55–0.99) and for sesame/flaxseeds (0.73, 95% CI=0.54–0.99; Table 5). There was no significant trend with increasing intake of any lignan-rich food (all *P*_Trend_>0.1). No association between lignan-rich foods and BCSS was observed (Table 5).

Sensitivity analysis by median time between diagnosis and FFQ completion (168 days, interquartile range=414 days) showed no significant heterogeneity between risk estimates for the estimated enterolignans, fibre, and lignan-rich foods and OS (all *P*_heterogeneity_>0.05). Exclusion of 19 phyto-oestrogen supplement users also did not alter the risk estimates for any variable in association with OS (data not shown).

In 2228 patients with early-stage (I–IIIa) disease, the HRs for overall mortality associated with estimated enterolignans, fibre, and lignan-rich foods were similar to that in the total study population (all *P*_heterogeneity_>0.1). The reductions in HR associated with the highest compared with the lowest quintiles were stronger for estimated enterolactone (HR=0.49, 95% CI=0.30–0.80, *P*_Trend_=0.02), enterodiol (HR=0.56, 95% CI=0.35–0.91, *P*_Trend_=0.02), and fibre (HR=0.45, 95% CI=0.26–0.80, *P*_Trend_=0.01).

There was no significant heterogeneity in the associations with estimated enterolignans and fibre by ER status (Supplementary Table 2), ER/PR status, and menopausal hormone therapy use at diagnosis (all *P*_heterogeneity_>0.1; data not shown).

## Discussion

Our data provide first evidence that postmenopausal patients with higher levels of the estimated enterolignans, enterolactone and enterodiol, may have a significantly increased OS, which is independent of ER status of the tumour. Higher dietary fibre intake was also associated with a significantly better survival after breast cancer. In addition, the lower mortality associated with higher enterolignan levels in women with lower than median fibre intake provides some evidence for an effect of enterolignans, which is independent of fibre intake.

The estimation of enterolignan – the bioactive forms in the human body – using the method by Thompson *et al* (1991), which is based on *in vitro* fermentation of various plant foods with human faecal bacteria, has several strengths. It indirectly accounts for all enterolignan precursors and covers the long-term exposure to enterolignans. Nevertheless, the results must be interpreted cautiously as the estimation does not account for intra- and inter-individual variation of gut bacteria and metabolism. Also, the estimation is based on data collected in the early 1990s but, to our knowledge, there are no other data available to estimate the intestinally produced amounts of enterolactone and enterodiol, and this approach was also used by a previous study (Touillaud *et al*, 2007).

Dietary intake of bread, vegetables, and fruits, which are important sources of lignans, was not individually associated with breast cancer prognosis. Intake of seeds, which are rich sources of lignans (Penalvo *et al*, 2005), was associated with a nonsignificantly decreased overall mortality, but there was no significant dose-response relationship. Of note is that seeds contain other food constituents, like *α*-linoleic acids (present in flaxseed), that have been shown to have anti-carcinogenic and anti-proliferative properties, which could render a protective effect (Wang *et al*, 2005; Bozan and Temelli, 2008).

So far, no other study has assessed the association between estimated enterolignans and breast cancer prognosis. Comparison of our findings with results on dietary lignan intake may not be appropriate. Nevertheless, in two smaller studies (each with ∼800 postmenopausal women) conducted in the United States, higher dietary lignan intake was associated with lower mortality after breast cancer in one (McCann *et al*, 2009) but not in the other (Fink *et al*, 2007). Lignan intake was very different in these studies – one reporting a median intake of 245 *μ*g day^–1^ (McCann *et al*, 2009) and the other reporting 10 times higher intake levels (0 to >9 mg day^–1^) (Fink *et al*, 2007). In line with our results, a recent Danish study observed a significantly increased survival with higher plasma enterolactone levels (Olsen *et al*, 2011).

Lignans have been found in high amounts in fibre-rich foods (e.g., cereals, wholegrains), and enterolactone concentrations were primarily explained by vegetable, rye, and fibre intake in Finnish men (Nurmi *et al*, 2010). In our study, the primary sources of estimated enterolignans were intake of bread (white, brown, and wholegrain), vegetables, and fruits, which are also rich sources of fibre. Fibre intake could affect breast cancer risk by several mechanisms, including stimulation of the intestinal microflora, reduction of the enterohepatic oestrogen circulation, and thereby reduction of oestrogen concentrations in the body (Adlercreutz, 2007; Sonestedt and Wirfält, 2010). Additionally, increased fibre intake was found to be related to lower serum oestradiol levels in women with breast cancer (Rock *et al*, 2004).

To account for potential confounding by fibre, we adjusted the multivariable regression models for estimated enterolignans and OS by fibre intake. High levels of estimated enterolignans were no longer significantly associated with overall mortality, probably because of their high correlation with fibre, which may have introduced collinearity in the multivariable models. Also, the association between dietary fibre and overall survival was no longer significant after adjustment for enterolactone. Adjustment for fibre intake was not found to affect the association of lignan intake with postmenopausal breast cancer risk in a study conducted in France (Touillaud *et al*, 2007). However, the observation of a significant association of estimated enterolignans with a better prognosis in those with low dietary fibre intake strengthens the hypothesis that enterolignans may influence the prognosis of postmenopausal breast cancer independent of fibre intake. Furthermore, it has been suggested that high lignan intake might be an indicator of a healthy lifestyle (Sonestedt and Wirfält, 2010). However, adjustment for lifestyle factors including physical activity, BMI, alcohol, and smoking did not affect the associations observed for enterolignans.

No significant effect heterogeneity by ER status of the tumours and menopausal hormone therapy use at diagnosis was observed for the association of estimated enterolignans and fibre with overall mortality, which is in line with the results of another study on dietary lignans and prognosis (Fink *et al*, 2007). Therefore, hormone-dependent and -independent mechanisms of action might be involved in the observed association with enterolignans. Enterolignans are able to bind to ERs, thereby preventing endogenous oestrogens from binding and inhibiting tumour growth stimulation (Adlercreutz and Mazur, 1997). In animal studies, several hormone-independent mechanisms have been suggested, such as inhibition of angiogenesis, tumour growth, and metastasis, as well as stimulation of apoptosis (Power *et al*, 2006; Saarinen *et al*, 2008, 2010). Moreover, an intervention with flaxseed in postmenopausal breast cancer patients resulted in an increased apoptosis and a decrease of tumour biological markers in tumour tissues (Thompson *et al*, 2005).

The strength of our study was the large population-based patient sample with complete follow-up and verification of causes of death using death certificates as well as the consideration of all relevant prognostic factors. Sensitivity analyses were performed, which showed that results were not affected by time of diagnosis and FFQ completion. Additionally, fibre intake, enterolignan levels, and lignan-rich foods (except for the seeds) were estimated using a validated dietary questionnaire. Thereby, a broad spectrum of dietary lignan sources was addressed. The FFQ assessed dietary habits over the entire 12 months before diagnosis and, thus, captures the long-term situation of enterolignan exposure.

In the interpretation of our results, we also need to address some limitations. The assessment of dietary intake may be subject to measurement errors that are not only due to recall bias but also to the estimation using food-composition databases, which may not be complete for the whole range of foods consumed. Dietary changes after diagnosis, which could have contributed to survival, were not accounted for in this analysis. Although we collected comprehensive data and assessed many possible confounding factors, residual confounding could not be entirely ruled out. Also, sample size was limited in subgroups and therefore results must be interpreted cautiously.

In conclusion, we found some evidence for a better prognosis in postmenopausal breast cancer patients who have high estimated enterolignan and dietary fibre exposures. The associations of estimated enterolignans with survival were independent of ER status of the tumour and may, in part, be independent of dietary fibre intake. However, further investigations on enterolignans measured in serum or urine and breast cancer prognosis are warranted to confirm our results and to disentangle the possible mechanisms involved.

## Figures and Tables

**Table 1:** Distribution of sociodemographic variables and prognostic factors for 2653 postmenopausal breast cancer patients by vital status

	**Alive**		**Deceased**		***χ* ^2^**
	***N***	**%**	***N***	**%**	***P*-value**
*Age at diagnosis (years)*					<0.01
50–54	170	7.3	22	6.9	
55–59	541	23.2	73	22.7	
60–65	760	32.6	80	24.9	
65–70	622	26.7	88	27.4	
70–74	239	10.3	58	18.1	
					
*Adult BMI (kg* *m*^*–2*^)					<0.01
<18.5	66	2.8	10	3.1	
18.5–25	1757	75.3	212	66.0	
25–30	438	18.8	80	24.9	
>30	71	3.0	19	5.9	
					
*Tumour size*					<0.01
<2 cm	1296	55.6	94	29.3	
2–5 cm	715	30.7	141	43.9	
⩾5 cm	61	2.6	22	6.9	
Growth into chest wall	36	1.5	28	8.7	
*In situ*	155	6.7	4	1.3	
Neoadjuvant chemotherapy	64	2.7	24	7.5	
Missing	5	0.2	8	2.5	
					
*Nodal status*					<0.01
0	1507	64.6	121	37.7	
1–3	455	19.5	95	29.6	
4–9	99	4.3	31	9.7	
⩾10	48	2.1	38	11.8	
*In situ*	155	6.7	4	1.3	
Neoadjuvant chemotherapy	64	2.7	24	7.5	
Missing	4	0.2	8	2.5	
					
*Metastasis*					<0.01
No	2145	92.0	263	81.9	
Yes	23	1.0	48	15.0	
*In situ*	155	6.7	4	1.3	
Missing	9	0.4	6	1.9	
					
*Grade*					<0.01
G1+G2	1589	68.1	148	46.1	
G3+G4	517	22.2	143	44.6	
*In situ*	155	6.7	4	1.3	
Neoadjuvant chemotherapy	64	2.7	24	7.5	
Missing	7	0.3	2	0.6	
					
*ER/PR status*					<0.01
ER+/PR+	1391	59.7	149	46.4	
ER+/PR− or ER−/PR+	411	17.6	59	18.4	
ER−/PR−	304	13.0	84	26.2	
*In situ*	155	6.7	4	1.3	
Neoadjuvant chemotherapy	64	2.7	24	7.5	
Missing	7	0.3	1	0.3	
					
*HER2-neu status*					<0.01
Positive	360	15.4	72	22.4	
Negative	1528	65.5	195	60.8	
*In situ*	155	6.7	4	1.3	
Neoadjuvant chemotherapy	64	2.7	24	7.5	
Missing	225	9.7	26	8.1	
					
*Breast cancer detection type*				<0.01	
Self-detected	1147	49.2	241	75.1	
Physician-detected	1175	50.4	80	24.9	
Missing	10	0.4	0	0.0	
					
*Menopausal hormone therapy use at diagnosis*				<0.01	
No/past	1151	49.4	226	70.4	
Yes	1165	50.0	93	29.0	
Missing	16	0.7	2	0.6	
					
*Diabetes*					<0.01
No	2158	92.5	278	86.6	
Yes	171	7.3	42	13.1	
Missing	3	0.1	1	0.3	
					
*Cardiovascular disease*					<0.01
No	1197	51.3	131	40.8	
Yes	1135	48.7	190	59.2	
					
*Surgery type*					<0.01
Breast ablation	263	11.3	76	23.7	
Breast conservation	775	33.2	76	23.6	
Missing	1294	55.5	169	52.7	
					
*Leisure time physical activity (since age 50)*				0.38	
<28 MET h per week	608	26.1	92	28.7	
⩾28 MET h per week	1701	72.9	229	71.3	
Missing	23	1.0	0	0.0	
					
*Phyto-oestrogen supplement use*			0.16		
No	2313	99.2	321	100.0	
Yes	19	0.8	0	0.0	
					
*Smoking*					0.75
Never	1276	54.7	175	54.5	
Former	648	27.8	85	26.5	
Current	408	17.5	61	19.0	
					
*Alcohol (g* *day*^*–1*^)					0.53
0	316	13.6	42	13.1	
<19	1719	73.7	231	72.0	
⩾19	296	12.7	48	15.0	
Missing	1	0.0	0	0.0	

Abbreviations: BMI=body mass index; ER=oestrogen receptor; HER2-neu=human epidermal growth factor receptor 2; PR=progesterone receptor; MET=metabolic equivalent value.

**Table 2:** Consumption levels of estimated enterolignans, dietary fibre, and lignan-rich foods by vital status

**Item**	**Vital status (*N*)**	**Mean**	**Median**	**IQR**	***P* Kruskal–Wallis**
Enterolactone (*μ*g day^–1^)	Alive (2332)	312.5	279.4	190.5	<0.001
	Deceased (321)	280.5	246.7	166.6	
					
Enterodiol (*μ*g day^–1^)	Alive (2332)	465.3	373.3	296.5	<0.001
	Deceased (321)	403.8	325.6	212.8	
					
Fibre (g day^–1^)	Alive (2332)	20.8	20.0	8.1	0.04
	Deceased (321)	20.0	19.6	8.2	
					
Sunflower-/pumpkinseeds (g day^–1^)	Alive (2332)	3.4	0.2	2.1	0.003
	Deceased (321)	2.3	0.2	2.1	
					
Sesame/flaxseeds (g day^–1^)	Alive (2332)	2.5	0.0	1.4	0.05
	Deceased (321)	1.7	0.0	1.4	
					
Bread (g day^–1^)	Alive (2332)	131.9	123.8	73.5	0.29
	Deceased (321)	136.4	130.7	76.5	
					
Vegetables (g day^–1^)	Alive (2332)	135.8	119.1	72.8	0.05
	Deceased (321)	127.1	113.2	73.1	
					
Fruit (g day^–1^)	Alive (2332)	165.3	130.3	117.2	0.03
	Deceased (321)	155.5	106.5	106.9	

Abbreviation: IQR=interquartile range.

**Table 3:** Multivariable^a^ HRs of estimated enterolignans, dietary fibre, and overall and breast cancer-specific mortality of postmenopausal breast cancer patients

			**Overall mortality**				**Breast cancer-specific mortality**			
**Q**	**Median levels (IQR) (*μ*g** **day**^–1^)	***N***	**Deaths**	**HR**	**(95% CI)**	***P*_Trend_**	**Breast cancer deaths**	**HR**	**(95% CI)**	***P*_Trend_**
*Enterolactone*										
1	146.0 (41.9)	530	84	1.00	Ref		59	1.00	Ref	
2	211.9 (28.6)	531	74	0.87	(0.62–1.22)		54	0.88	(0.59–1.32)	
3	274.3 (32.8)	530	53	0.69	(0.48–1.00)		38	0.77	(0.50–1.20)	
4	360.7 (50.4)	530	62	0.91	(0.63–1.32)		48	1.18	(0.76–1.82)	
5	502.0 (142.5)	532	48	0.60	(0.40–0.89)	0.02	36	0.69	(0.43–1.10)	0.35
										
*Enterodiol*										
1	186.9 (59.8)	530	76	1.00	Ref		54	1.00	Ref	
2	279.8 (39.5)	531	76	0.95	(0.67–1.33)		53	0.92	(0.61–1.39)	
3	366.8 (50.2)	530	70	0.97	(0.69–1.37)		53	1.07	(0.72–1.61)	
4	499.9 (89.0)	531	53	0.79	(0.54–1.15)		37	0.84	(0.53–1.33)	
5	857.5 (368.6)	531	46	0.63	(0.42–0.95)	0.02	38	0.81	(0.51–1.29)	0.40
										
*Fibre*										
1	13.3 (3.0)^b^	530	80	1.00	Ref		55	1.00	Ref	
2	16.9 (1.5)^b^	531	62	0.71	(0.50–1.02)		42	0.74	(0.48–1.16)	
3	19.9 (1.5)^b^	531	68	0.68	(0.47–0.97)		56	0.82	(0.54–1.26)	
4	23.2 (1.9)^b^	530	53	0.64	(0.43–0.95)		41	0.71	(0.44–1.14)	
5	28.9 (5.7)^b^	531	53	0.52	(0.32–0.82)	0.01	41	0.64	(0.37–1.11)	0.01

Abbreviations: CI=confidence interval; ER=oestrogen receptor; HR=hazard ratio; IQR=interquartile range; PR=progesterone receptor; Q=quantile.

a Stratified by age at diagnosis. Adjusted for tumour size, nodal status, metastasis, grade, ER/PR status, breast cancer detection type, diabetes, menopausal hormone therapy use at diagnosis, study centre, and energy intake.

b In g day^–1^.

**Table 4:** Multivariable^a^ HRs of enterolignans and overall mortality of postmenopausal breast cancer patients by dietary fibre intake

	**Low fibre intake** b			**High fibre intake** b		
**Q**	**HR**	**(95% CI)**	***P*_Trend_**	**HR**	**(95% CI)**	***P*_Trend_**
*Enterolactone*						
1	1.00	Ref		1.00	Ref	
2	0.82	(0.56–1.20)		2.07	(0.67–6.46)	
3	0.65	(0.40–1.07)		1.39	(0.48–4.03)	
4	0.84	(0.44–1.58)		2.04	(0.71–5.18)	
5	0.32	(0.10–1.04)	0.03	1.25	(0.44–3.58)	0.08
						
*Enterodiol*						
1	1.00	Ref		1.00	Ref	
2	0.91	(0.63–1.33)		1.48	(0.50–4.42)	
3	0.90	(0.57–1.42)		1.50	(0.54–4.16)	
4	0.39	(0.16–0.99)		1.58	(0.57–4.37)	
5	0.48	(0.21–1.07)	0.01	1.08	(0.38–3.04)	0.05

Abbreviations: CI=confidence interval; ER=oestrogen receptor; HR=hazard ratio; PR=progesterone receptor; Q=quantile.

a Stratified by age at diagnosis. Adjusted for tumour size, nodal status, metastasis, grade, ER/PR status, breast cancer detection type, diabetes, menopausal hormone therapy use at diagnosis, study centre, and energy intake.

b Below *vs* equal to or above the median dietary fibre intake.

**Table 5:** HRs of phyto-oestrogen-rich foods and overall and breast cancer-specific mortality of postmenopausal breast cancer patients

**Food item**	**Survival measure**		**No consumption**	**Low consumption** a	**High consumption** a	***P*_Trend_**
Sunflower/pumpkinseeds	Overall mortality	*N*/deaths (median intake)	1126/157 (0 g day^–1^)	747/82 (0.4 g /day^–1^)	780/82 (4.5 g day^–1^)	
		Multivariable HR (95% CI)^b^	1.00 (Ref)	0.74 (0.55–0.99)	0.87 (0.66–1.15)	0.17
	BC mortality	Multivariable HR (95% CI)^b^	1.00 (Ref)	0.88 (0.63–1.24)	1.12 (0.79–1.57)	0.35
						
Sesame/flaxseeds	Overall mortality	*N*/deaths (median intake)	1355/182 (0 g day^–1^)	591/57 (0.3 g /day^–1^)	707/82 (3.6 g /day^–1^)	
		Multivariable HR (95% CI)^b^	1.00 (Ref)	0.69 (0.50–0.95)	0.90 (0.68–1.19)	0.26
	BC mortality	Multivariable HR (95% CI)^b^	1.00 (Ref)	0.75 (0.51–1.09)	1.21 (0.87–1.68)	0.87
						
			**Tertile 1**	**Tertile 2**	**Tertile 3**	
Bread	Overall mortality	*N*/deaths (median intake)	875/98 (80 g day^–1^)	902/118 (125 g day^–1^)	876/105 (191 g day^–1^)	
		Multivariable HR (95% CI)^b^	1.00 (Ref)	1.50 (1.12–2.01)	1.31 (0.93–1.83)	0.07
	BC mortality	Multivariable HR (95% CI)^b^	1.00 (Ref)	1.38 (0.98–1.94)	1.10 (0.74–1.63)	0.33
						
Vegetables	Overall mortality	*N*/deaths (median intake)	875/118 (79 g day^–1^)	902/100 (118 g day^–1^)	876/103 (183 g day^–1^)	
		Multivariable HR (95% CI)^b^	1.00 (Ref)	0.97 (0.72–1.30)	1.09 (0.80–1.48)	0.56
	BC mortality	Multivariable HR (95% CI)^b^	1.00 (Ref)	0.86 (0.61–1.22)	1.01 (0.70–1.46)	0.16
						
Fruits	Overall mortality	*N*/deaths (median intake)	876/122 (79 g day^–1^)	902/104 (128 g day^–1^)	876/95 (259 g day^–1^)	
		Multivariable HR (95% CI)^b^	1.00 (Ref)	0.90 (0.68–1.20)	0.84 (0.61–1.16)	0.80
	BC mortality	Multivariable HR (95% CI)^b^	1.00 (Ref)	0.90(0.64–1.27)	0.86 (0.59–1.25)	0.82

Abbreviations: BC=breast cancer-specific; CI=confidence interval; ER=oestrogen receptor; HR=hazard ratio; PR=progesterone receptor.

a Low and high consumption defined by below and above/equal to median consumption of the consumers, respectively.

b Stratified by age at diagnosis. Adjusted for tumour size, nodal status, metastasis, grade, ER/PR status, breast cancer detection type, diabetes, menopausal hormone therapy use at diagnosis, study centre, and energy intake.

^c^Stratified by age at diagnosis.
