# Supplementary material for: Estimated enterolignans, lignan-rich foods, and fibre in relation to survival after postmenopausal breast cancer
Source: Br J Cancer. 2011 Sep 13;105(8):1151–7. doi: 10.1038/bjc.2011.374 (PMC3208499; doi:10.1038/bjc.2011.374)
Supplement: Supplementary Information [file bjc2011374x1.doc]

**Supplementary Table 1. Multivariable^a^ hazard ratios (HRs) of enterolignans, dietary fibre and overall mortality of postmenopausal breast cancer patients with additional adjustments**

|  | **Q** | **N** | **Deaths** | **HR** | **(95% CI)** | **P_Trend_** |
| --- | --- | --- | --- | --- | --- | --- |
| **Enterolactone^b^** | 1 | 530 | 84 | 1.00 | Ref |  |
|  | 2 | 531 | 74 | 0.92 | (0.65-1.31) |  |
|  | 3 | 530 | 53 | 0.77 | (0.51-1.15) |  |
|  | 4 | 530 | 62 | 1.05 | (0.68-1.64) |  |
|  | 5 | 532 | 48 | 0.75 | (0.43-1.31) | 0.42 |
|  |  |  |  |  |  |  |
| **Enterodiol^b^** | 1 | 530 | 76 | 1.00 | Ref |  |
|  | 2 | 531 | 76 | 1.00 | (0.70-1.42) |  |
|  | 3 | 530 | 70 | 1.07 | (0.74-1.55) |  |
|  | 4 | 531 | 53 | 0.91 | (0.59-1.42) |  |
|  | 5 | 531 | 46 | 0.78 | (0.47-1.29) | 0.21 |
|  |  |  |  |  |  |  |
| **Fibre^c^** | 1 | 530 | 80 | 1.00 | Ref |  |
|  | 2 | 531 | 62 | 0.74 | (0.51-1.07) |  |
|  | 3 | 531 | 68 | 0.73 | (0.49-1.08) |  |
|  | 4 | 530 | 53 | 0.72 | (0.46-1.13) |  |
|  | 5 | 531 | 53 | 0.64 | (0.35-1.18) | 0.39 |

Abbreviations: CI, confidence interval, ER, oestrogen receptor, HR, hazard ratio, PR, progesterone receptor, Q, quantile.

^a^ Stratified by age at diagnosis. Adjusted for tumour size, nodal status, metastasis, grade, ER/PR status, breast cancer detection type, diabetes, menopausal hormone therapy use at diagnosis, study centre, and energy intake.

^b^ Additionnally adjusted for total dietary fibre intake.

^c^ Additionally adjusted for estimated enterolactone levels.

**Supplementary Table 2. Multivariable^a^ hazard ratios (HRs) of estimated enterolignans, dietary fibre and overall mortality of postmenopausal breast cancer patients stratified by estrogen receptor (ER) status**

|  |  |  | **ER-positive tumors** | | |  |  |  | **ER-negative tumors** | | |  |
| --- | --- | --- | --- | --- | --- | --- | --- | --- | --- | --- | --- | --- |
|  | **Q** | **N** | **Deaths** | **HR** | **(95% CI)** | **P_Trend_** |  | **N** | **Deaths** | **HR** | **(95% CI)** | **P_Trend_** |
| **Enterolactone** | 1 | 403 | 56 | 1.00 | Ref |  |  | 93 | 25 | 1.00 | Ref |  |
|  | 2 | 390 | 58 | 1.26 | (0.84-1.90) |  |  | 103 | 14 | 0.45 | (0.21-0.96) |  |
|  | 3 | 390 | 31 | 0.65 | (0.40-1.05) |  |  | 104 | 22 | 0.94 | (0.50-1.77) |  |
|  | 4 | 394 | 36 | 0.92 | (0.57-1.49) |  |  | 97 | 26 | 1.27 | (0.66-2.46) |  |
|  | 5 | 389 | 32 | 0.76 | (0.46-1.25) | 0.14 |  | 92 | 16 | 0.52 | (0.24-1.11) | 0.09 |
|  |  |  |  |  |  |  |  |  |  |  |  |  |
| **Enterodiol** | 1 | 400 | 49 | 1.00 | Ref |  |  | 99 | 24 | 1.00 | Ref |  |
|  | 2 | 406 | 62 | 1.18 | (0.78-1.78) |  |  | 88 | 13 | 0.59 | (0.27-1.29) |  |
|  | 3 | 385 | 41 | 0.97 | (0.62-1.51) |  |  | 106 | 29 | 1.10 | (0.59-2.06) |  |
|  | 4 | 396 | 31 | 0.70 | (0.43-1.16) |  |  | 96 | 21 | 1.08 | (0.55-2.12) |  |
|  | 5 | 379 | 30 | 0.78 | (0.47-1.29) | 0.29 |  | 100 | 16 | 0.53 | (0.25-1.14) | 0.02 |
|  |  |  |  |  |  |  |  |  |  |  |  |  |
| **Fibre** | 1 | 406 | 58 | 1.00 | Ref |  |  | 85 | 19 | 1.00 | Ref |  |
|  | 2 | 382 | 42 | 0.61 | (0.39-0.96) |  |  | 110 | 20 | 1.07 | (0.51-2.26) |  |
|  | 3 | 388 | 42 | 0.53 | (0.34-0.82) |  |  | 99 | 25 | 1.41 | (0.67-2.98) |  |
|  | 4 | 392 | 36 | 0.55 | (0.34-0.89) |  |  | 102 | 21 | 0.98 | (0.43-2.20) |  |
|  | 5 | 398 | 35 | 0.47 | (0.27-0.82) | 0.05 |  | 93 | 18 | 0.92 | (0.36-2.39) | 0.33 |

Abbreviations: CI, confidence interval, ER, oestrogen receptor, HR, hazard ratio, Q, quantile.

^a^ Stratified by age at diagnosis. Adjusted for tumour size, nodal status, metastasis, grade, breast cancer detection type, diabetes, menopausal hormone therapy use at diagnosis, study centre, and energy intake.
